# Medical student experiences of equality, diversity, and inclusion: content analysis of student feedback using Bronfenbrenner’s ecological systems theory

**DOI:** 10.1186/s12909-023-04986-8

**Published:** 2024-01-03

**Authors:** Helen Anne Nolan, Katherine Owen

**Affiliations:** University of Warwick, Coventry, CV4 7AL UK

**Keywords:** Equality, diversity and inclusion, Inclusive education, Student feedback, Ecological systems theory

## Abstract

**Background:**

Issues relating to equality, diversity, and inclusion (EDI) significantly impact on medical student achievement and wellbeing. Interventions have been introduced at curricular and organisational levels, yet progress in addressing these issues remains limited. Timely evaluation is needed to assess effectiveness of interventions, and to explore issues and interactions in learning environments impacting on student experience. We introduced an anonymous question concerning students’ experiences of EDI into routine online student feedback questionnaires, to scope the nature of ongoing issues and develop greater understanding of students’ experiences in our programme environment. Ecological systems theory, which conceptualizes learning as a function of complex social interactions, determined by characteristics of individual learners and their environment, provides a framework for understanding.

**Methods:**

Free-text responses regarding experiences of EDI gathered over 20 months from all programme years (*n* = 760) were pooled for analysis, providing a holistic overview of experiences in the learning environment. A counting exercise identified broad categories reported by students. Content analysis of the qualitative dataset was undertaken. Bronfenbrenner’s ecological systems theory was applied as a framework to demonstrate interdependencies between respondents’ experiences and environments, and associated impacts.

**Results:**

Three hundred and seventy-six responses were received relating to wide-ranging EDI issues, most frequently gender or ethnicity. Responses mapped onto all areas of the ecological systems model, with frequent links between subsystems, indicating considerable complexity and interdependencies. Interpersonal interactions and associated impacts like exclusion were frequently discussed. Differential experiences of EDI-related issues in medical school compared to clinical settings were reported. Impacts of institutional leadership and wider societal norms were considered by respondents. Respondents discussed their need for awareness of EDI with reference to future professional practice.

**Conclusions:**

Implementation of a regular free-text evaluation question allowed data-gathering across cohorts and throughout several stages of the curriculum, illuminating student experience. Connections established demonstrated intersectionality, and how environment and other factors interact, impacting on student experiences. Students experience EDI-related issues on multiple levels within the educational environment, with consequent impacts on learning. Any successful approach towards tackling issues and promoting equity of opportunity for all requires multi-level actions and widespread culture change. Students can offer fresh and distinct perspectives regarding change needed, to complement and diversify perspectives provided by staff and organisational leadership. Student voice should be enabled to shape change.

**Supplementary Information:**

The online version contains supplementary material available at 10.1186/s12909-023-04986-8.

## Introduction

In response to systematic injustices and inequalities experienced by minoritized groups (a definition based on power and fairness, not numbers) [1], actions to redress these issues have gained momentum, often under the banner of equality, diversity, and inclusion (EDI) in European settings [2, 3], with related but distinct nomenclature (diversity, equity and inclusion, DEI) in use in other geographical settings [4, 5]. Recommendations for addressing EDI issues in curricula and training acknowledge that factors underpinning inequitable and exclusionary practices are regularly subtle, in the form of microaggressions [6] and other artefacts of hidden curricula [7–9]. Practices may be structurally ingrained via policy, reflecting norms of dominant groups [10]. Poorer assessment outcomes experienced by racially minoritized students compared to white counterparts are not accounted for by differences in ability, but instead differences in relationships with peers and trainers, and differences in the learning environment [11–13]. Similar awarding gaps affect students experiencing disability [14, 15]. Organisations are urged to harness student voice to expose and understand student-identified barriers to inclusion and to regularly evaluate effectiveness of interventions [4, 16, 17]. While student dialogue is encouraged, caution is recommended against overburdening only minoritized students with identifying solutions to issues raised, a condition known as the “minority tax” [18]. As remediative actions advance, organisations must enable capturing of representative student voice, remain open to recognising EDI issues, ensure proposed solutions can enhance curricula, learning environments and healthcare outcomes, and support belonging for all groups.

Professional sector guidance advocates various approaches to addressing EDI issues including anti-racist pedagogies, and inclusion by design [2, 19–22]. Effective operationalisation requires focus on institutional accountability and governance, student and staff recruitment, curriculum design and delivery, and data monitoring and evaluation [2]. Ongoing evaluation is recommended to ensure effectiveness of interventions and to identify emergent issues [16, 17]. Recommendations emphasise monitoring data relating to protected characteristics or class as defined in equalities legislation, relying on quantitative metrics for assurance and with a singular focus on “diversity”—the presence and celebration of perceived differences [4], moving beyond purely visible features and including demographic features e.g. socioeconomic features [23]. Equality refers to fairness, ensuring that individuals or groups are not treated less favourably because of protected characteristics [24], an entity that in itself has limitations and is superseded by equity, which emphasizes equality of opportunity and resource, while providing more to those with greater needs [24, 25]. Inclusion seeks to ensure that everyone is valued and welcomed, in an environment where individuals are offered fair opportunities and enabled to contribute [24]. Issues of EDI in environments and curricula do not exist in isolation, but instead intersect in complex ways [25]. Addressing diversity metrics by widening access to medical study in the absence of inclusive policies and organisational culture or equitable resource allocation indicates lack of strategic direction. This approach overlooks other important interdependencies, sustaining disadvantage experienced by minoritised groups [26]. The rapid pace of attempts to address issues relating to EDI in healthcare professions education may result in a dearth of evidence-based, practical strategy to optimally operationalise actions [27]. Equality, diversity, and inclusion may be subject to attempted disaggregation, with failure to recognise these interdependencies. Efforts frequently have been misdirected to addressing these entities individually, and in isolation from each other. Authors have cautioned against this “reductionist approach” to EDI which may hinder transformational and sustainable change in education. There are growing calls for recognition and exploration of these interdependent and inextricably linked entities in order to create an effective and holistic system of change, [2, 28].

Just as EDI in education should not be subjected to disaggregation in developing understanding [4], neither should issues be explored in a decontextualised manner. Consideration of contextual relevance and authenticity is required [27]. Education itself is a social process, with learning and learners situated in complex environments. Social ecological theory, which highlights the complexity of interactions between individual, institutional, social, and cultural elements [29], has been applied to processes of human development and learning environments [30, 31]. This theory demonstrates the nested nature of the individual and their experiences within the learning environment [30, 32]. Applied by Bronfenbrenner to education contexts and processes [30, 33], social ecological theory led to identification of key factors influencing student learning including; 1) the learner’s own characteristics and identity, and the environments in which they exist, 2) and the relationships and interconnections between them [33]. This resulted in development of Ecological Systems Theory (EST) as a framework to identify and organise salient factors within different environments and levels, and to demonstrate the relationships and interconnections between them. This theoretical framework centralises the role of the learner and describes five subsystems that interact and contribute to the process of learning and learning environments, demonstrating interdependent relationships and bidirectional action across these subsystems. At the individual level, learners construct meaning relative to others and to shared beliefs. Simultaneously, individuals influence their social ecology by contributing to practice and culture across multiple subsystems [29, 32]. Learning environments are also subject to wider political and societal drivers as healthcare classrooms are not buffered from these influences but may in fact replicate and sustain them [34]. EST can be used to study single factors, groups of factors or whole systems as well as the relationships therein. EST has been used to inform understanding of ecologies of inclusive education in schools [30]. Despite momentum gained in attempting to advance EDI in medical education organisations and environments, no such systematic ecological exploration of student experiences of EDI in medical education has been identified.

In this paper we describe the introduction of an open-ended free-text question, as part of regular student evaluation of teaching questionnaires, to explore students’ experiences of any issues identified by them as relating to EDI on a graduate-entry medical programme. Bronfenbrenner’s conceptual framework was applied to make sense of human interactions and development within this socially constructed medical education environment or ecology. This holistic model was chosen to support categorisation of factors experienced as salient by learners,  to illustrate interactions and relationships between factors and associated impacts, and to capture the complexity of these interactions. This model was used to support exploration of the following research questions;

What are students’ experiences of the learning environment and curricular issues relating to EDI throughout the course of a graduate entry medical degree programme?

What influencing factors in the curriculum and learning environment in relation to EDI impact on students’ experiences?

Can offering students regular opportunities to provide anonymized free-text feedback identify underrecognised student-facing issues relating to EDI?

## Methods

### Context

Our four-year graduate-entry medical degree programme admits degree-holders from any academic background, thereby widening participation in medical education by traditionally underrepresented groups and in relation to student sociodemographic profile [35]. A wide range of action relating to differential awarding and EDI has been undertaken including faculty development and student training regarding active racial awareness and being active bystanders. Case-based learning is a signature pedagogy on this programme and here cases have been reviewed in partnership with students and updated to ensure representativeness. Concordant mentorship is offered to minoritized students. Student action groups have been established, and these include Student BME Network, Student Disability Network and Student LGBTQIA + Network. Programme staff and students have researched aspects of inclusive education [13, 15]. Metrics in relation to gender, racially minoritized and disabled student selection and attainment are routinely monitored. These various strands of activity are overseen by student-staff working groups.

### Participants and evaluation process

All students on the programme complete anonymous online feedback at the end of each module or “block” of teaching and clinical placement. At completion, students are informed that all feedback scores and comments are analysed independently and reported by the school’s education quality team and reviewed by academic year leads and clinical education teams. Reports are discussed at academic year management group meetings, which are attended by student representatives. These reports and discussions inform programme quality assurance and enhancement. Summaries and action plans are then shared with all students.

### Data collection

**Fig. 1:**
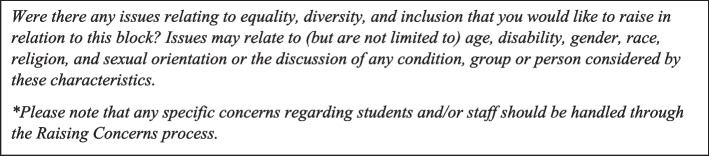
Open-ended free-text evaluation question

In September 2020, to coincide with ongoing action to address differential awarding and EDI, we introduced an open-ended free-text question (Fig. 1) into the feedback form for all cohorts, asking whether students had experienced any issues related to equality, diversity, and inclusion during that block. Data was sought here to inform better understanding of student experiences relating to EDI in the curriculum and learning environment and to identify hitherto unrecognized issues relating to EDI experienced by students. In developing the evaluation question, a range of educator and student stakeholders (including minorized student networks and other advocacy groups) were consulted, and their feedback was implemented. The term equality and the broader construct of EDI were used as these are the constructs upon which organisational policies and sector strategies are built in this region [3]. Expectations of conditions guaranteeing equality are enshrined in UK law [36] and, while recognising that equity is a necessary precondition for true equality, EDI terminology is readily recognised by stakeholders and learners.

Suggestions cited in the question included nine characteristics protected in legislation [36], but it noted that issues were not limited to these categories.

Where comments highlight issues requiring remediation or investigation, these are managed on a case-by-case basis by programme leadership and, where relevant, clinical education leadership. Positive feedback highlighting exemplary good practice is shared via course and academic quality management groups.

After 20 months, at completion of two academic years of use of this question, we undertook a qualitative content analysis of medical student experiences of EDI; we pooled and analysed the comments received, seeking themes and overarching issues relating to students’ experiences of EDI in the learning environment and curriculum.

### Ethics approvals

All students were contacted via digital message outlining the purpose of this analysis of pooled comments from a range of cohorts. Correspondence explained that the data was being reviewed for internal assurance and enhancement purposes. As this is a novel approach to student engagement in addressing EDI-related issues, it was explained there may be value in sharing our findings more widely in the healthcare education sector. Noting difficulties associated with confirming written consent from such large numbers of students and anonymized nature of the comments for analysis, students with any questions or concerns regarding the nature of the study or inclusion of their data were invited to contact either the researchers or the research governance team in confidence and contact details were shared in this correspondence. The study was reviewed and approved by University of Warwick Biomedical Sciences Research Ethics Committee.

### Data analysis

All responses to this question received since its inception in September 2020, were collated onto a Microsoft Excel spreadsheet and labelled according to the block on which they were received. Each comment was assigned a unique identifier number.

An initial counting exercise of the dataset was undertaken to scope out the broad nature and categories, and to summarise the core issues commented upon [37, 38]. Content analysis of the dataset was undertaken to further explore these broad categories reported [39]; researchers undertook an initial coding exercise on the same selection of 20 comments from a range of different blocks to identify preliminary codes and establish consensus between researchers. Coding of data here was inductive, driven by respondents’ comments and experiences that they reported [40]. After independent completion of this exercise, we reconvened to discuss initial codes and to assess researcher agreement. Here discrepancies were discussed in relation to codes applied and new codes identified, and agreement was reached through discussion and negotiation. This process was then completed for a second sample, once again identifying further codes, and allowing researchers opportunity for further calibration. We reconvened again and updated the codebook and discussed areas of inconsistency or disagreement (Codebook available, Appendix 1).

Researchers then coded the full dataset using the codes established and with the option to add new codes, allowing familiarization and immersion in the data. Researchers maintained reflective journals capturing observations and uncertainties and proposed new codes. Regular researcher meetings occurred throughout the analysis and reflective journals and discrepant views were discussed. Additional perspectives from student groups and staff with leadership roles in inclusive education were also sought. These measures allowed agreement to be established through perspective taking, discussion and negotiation.

**Fig. 2:**
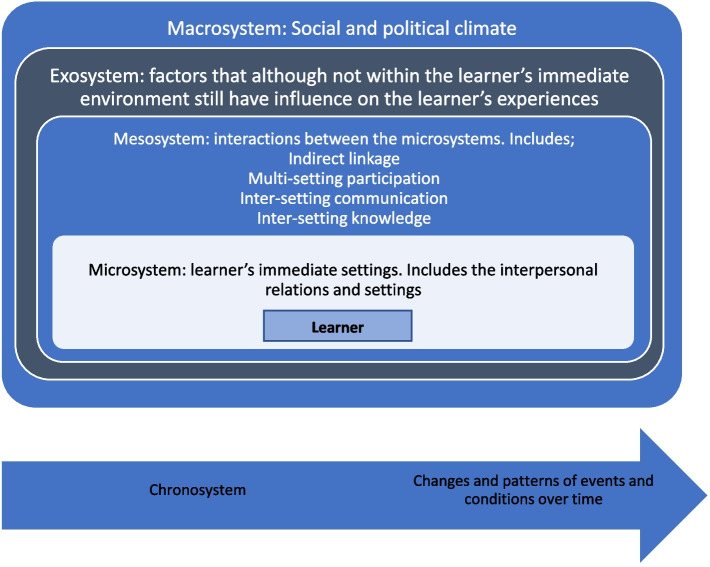
Bronfenbrenner’s ecological systems model

Once all data were coded, we applied Bronfenbrenner’s theory (Fig. 2, Table 1) to make sense of interactions and relationships within this socially constructed medical education environment or ecology. This conceptual framework was employed to map codes and identify themes, demonstrating connections between issues and experiences identified as significant by respondents.

**Table 1:** Level's of Bronfenbrenner's ecological systems theory [30–33]

Learner; individual level	The individual learner occupies a central position within the framework. The learner may be influenced by factors existing at and between each of the other levels
Micro-system	This level sits directly around the learner and contains the environment and the factors that the learner directly experiences. Factors here may include direct experiences with peers, faculty, patients and in the learning environment and experiences of the curriculum
Meso-system	This level acknowledges that factors within the micro-system are not isolated from each other. Dynamic relationships and interdependencies exist between the microsystem factors [31]. The mesosystem refers to the connections and relationships between two or more microsystems Bronfenbrenner identified four types of interactions that occur between microsystems factors and within the mesosystem that may impact on learners [31]
	**Multi-Setting Participation** The multi-setting participation link arises when the student engages in a more than one setting. This is relevant to medical student learning experiences due to their participation in a range of learning environments and settings including the medical school and a variety of placement, clinical and community settings
	**Indirect Linkage** In indirect linkage, the learner is impacted indirectly by connections between microsystems factors
	**Intersetting Communications** This includes communications and messages transmitted from one setting to another in order to provide “specific information to persons in the other setting” [31, 41] Communications here may be unidirectional or bidirectional and may occur in person, verbally, electronically etc
	**Inter-Setting Knowledge** This refers to the information or experience that exists in one setting about the other setting [41]
Exo-system	This level includes factors that are not directly within the learner’s immediate environment, however, still have influence on their experience. Factors may be those in the wider local context and may include policy and processes in medical school environment, including the programme leadership, strategy and policy, wider university leadership and policy, and culture and values
Macro-system	This level includes factors existing outside the medical learning environment (beyond the medical school, university, or clinical learning environment), but that influence the inner “sublevels” of the framework and the learner This may include the wider context in which the school exists, including social, political, historical, and global, as well as other factors, such as professional regulatory or curricular requirements
Chrono-system	This level considers impacts and changes associated with the passage of time and how this may influence the learner and their development over time

### Reflexivity, positionality

HN is the school’s Director of Education Quality. HN reviews all student feedback comments and reports and is a member of education quality management groups on the MBChB programme. HN is a cisgender woman and from a minority white ethnic group.

KO is the Director of Medical Studies and chair of the program’s learning and teaching quality committee. KO reviews all student feedback comments and reports, as well as comments received via the separate reporting concerns process, and is also a member of education quality management groups on the MBChB programme. KO is a cisgender woman and white British.

As part of negotiating own positionality and identities, we actively sought additional perspectives in analysis and sense making. Our Faculty Chair for EDI and student advocacy groups reviewed the data and our interpretations and report to assess appropriateness. Feedback from these checking stages was implemented. We triangulated our findings with quarterly reports from the school’s raising concerns process. This anonymous reporting process [42] is open to all students to raise concerns about unsafe, unprofessional, or other concerns experiences in medical school or clinical environments. Quarterly summaries of the number and types of concerns raised and overarching themes, as well as an overview of remediative actions implemented, are shared with students and faculty to promote organisational learning and to provide assurance. These summary reports provided the basis for triangulation, allowing us to assess similarities and differences in the nature of the issues being reported.

## Results

Three hundred and seventy-six comments were received between September 2020 and July 2022. The program has a total of 760 students across 3 phases over 4 years of study. Due to anonymity of data and the repeated opportunity to respond, it is not possible to determine the total number of students responding. A summary categorising the nature of responses is shown in Table 2, demonstrating the weighting of different categories (% of total comments for each Phase). Some comments were generic and were not possible to categorize, or covered more than one topic, hence some rows do not add up to total.

**Table 2:** Summary of nature of responses and issues raised in students’ responses

Programme Phase (Year)	Gender	Ethnicity	Sexuality	Disability	Religion	Socioeconomic status	Weight stigma
Phase 1 (Year 1, preclinical)	35 (17.4%)	108 (53.7%)	17 (8.5%)	29 (14.1%)	2 (< 1%)	4 (< 1%)	6 (< 1%)
Phase 2 (Year 2, clinical)	14 (22.2%)	13 (20.6%)	4 (6.3%)	13 (20.6%)	3 (4.8%)	0	0
Phase 3 (Years 3 and 4, clinical)	17 (27.4%)	14 (22.6%)	8 (11.3%)	3 (4.8%)	5 (8.1%)	2 (3.2%)	0

**Fig. 3:**
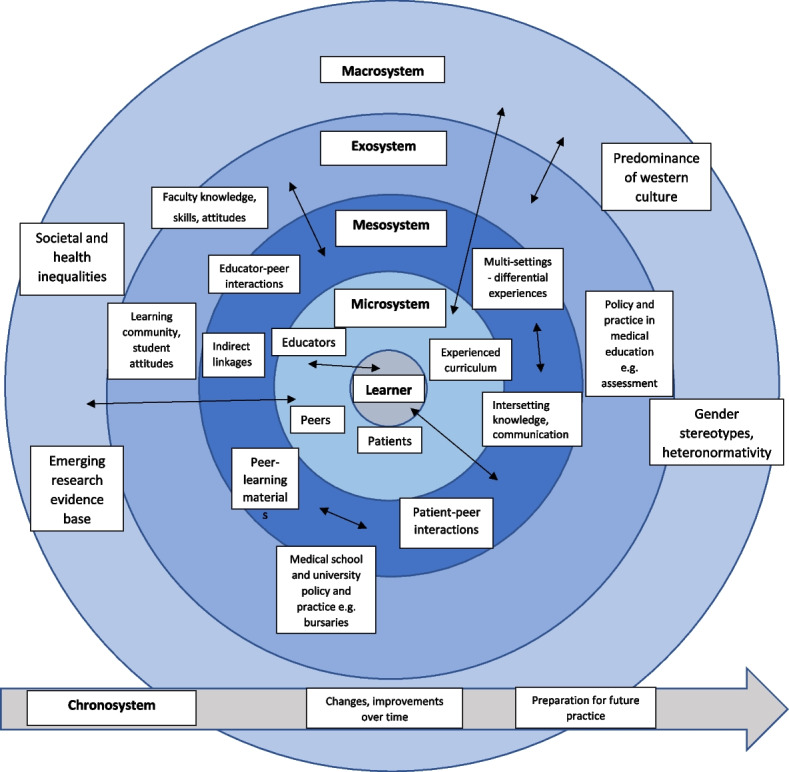
Themes and subthemes identified and mapped according to Bronfenbrenner’s ecological systems model

Having identified broad categories of issues, we then considered the relationship between various factors and the indicated impacts of these issues. Codes and themes were mapped onto the framework as shown in Fig. 3.

Respondents’ comments related to and demonstrated interaction between the five subsystems.

### Microsystem

The microsystem consists of the learner’s immediate settings and includes the interpersonal relations and settings of that individual [31].

#### Interpersonal interactions and relationships with peers, patients, and clinicians

Learners commented on a wide range of curricular and environmental features that they experienced and that impacted on them personally. The main theme in this domain related to direct interpersonal interactions and relationships between the respondent and their peers, educators, and patients. These relationships could be supportive or negative. Experiences discussed in individual learners’ comments were often mediated by learners’ own declared minoritized characteristics. Characteristic of comments in this theme were the use of language indicating how these situations made students feel: “excluded”, “ignored” or “frustrated”.

A common feature across facilitators, teaching staff and clinicians was reluctance to use an individual’s given name or failure to use this correctly and the personal impacts associated with this.

“…*started off the session with "I'm only going to call on people’s names I can pronounce", and then went on to only calling on the traditional white British names. The facilitator made no attempt to include ethnically diverse students into that session….I personally left that session feeling excluded.”*

There were also frequent perceived examples of staff (often clinicians) treating minoritized respondents differently.

*“It was widely noted that the consultant would only direct teaching to the male in the room, ignoring me (the female). This went as far as answering my questions to my male clinical partner.”*

Impactful direct interactions with patients were described infrequently, but predominantly negatively;

“*Patients asking where I am 'really from'. It has happened a couple of times now…it is annoying and rude.”*

Difficult interactions with peers were characterized by a perceived lack of knowledge and understanding of a minoritised characteristic and associated impacts.

*“I do not feel for a second any student would do this intentionally to undermine another, I do think it is somewhat disheartening as I feel I could get the answer if I had more time but couldn't process the question quick enough due to my dyslexia.”*

Other comments described positive, supportive interactions with peers;

*“There were no issues as everyone participated equally….No one was made to feel different or left out, it was a very inclusive community that I was glad to be a part of.”*

There were also instances of microaggressions due to reactions to a respondents’ outward appearance, which was interpreted by clinical staff members as remarkable, leading to student respondents experiencing differential treatment;

*“Some doctors and staff were noticeably colder to me due to me wearing hijab.”*

*“A white woman who was a staff member touched my braids.”*

#### Experiences of the curriculum and learning content

The second broad theme concerns the experienced curriculum. Many respondents appreciated the changes to content underway and suggested further enhancements that could be made.

“*I would like to say that the dermatology session was better this year in terms of including other skin tones so that was good.”*

However, there were examples of where the delivered content was not ethnically diverse or was stereotypical. This was particularly true for dermatology teaching where the lack of images on a range of skin tones was noted multiple times.

Accessibility of the curriculum remained an issue for students, particularly those with specific learning differences and sensory impairments. Late organisation of timetables impacted students with caring responsibilities and neurodivergent students disproportionately.

*“Some CBL slides are really text-heavy, so when it's the turn of myself with dyslexia, it puts a lot of stress and embarrassment when I stumble over the text.”*

A small number of tentative recommendations expressing uncertainly or making tentative recommendations were also received, suggesting this question had prompted respondents to reflect;

*“Maybe just the use of female models in anatomy/ clinical skills…Nothing particularly just possible ideas.”*

### Mesosystem

The mesosystem consists of interactions between the microsystems, highlighting that microsystems do not exist in isolation, but that there are important interrelations between them, and that microsystems influence each other. Bronfenbrenner identified four types of interactions and links that occur between microsystems and within the mesosystem that learners may experience; multi-setting participation, indirect linkage, inter-setting communications and inter-setting knowledge [31] (Fig. 2, Table 1).

#### Indirect linkage; indirect impacts and exclusions due to actions of others

Students are clearly impacted by the indirect actions of others, including clinicians, teaching staff and peers.

Several instances of clinical staff appearing, from the respondent’s perspective, to be more engaged with other learners were noted in the data; these were attributed to personal characteristics. This resulted in poorer learning experiences and feelings of frustration and exclusion.

*“I felt teaching was directed more at my clinical partner (a male), it's not the first time this has happened at med school.”*

Individual educators’ expressed views regarding patient groups also impacted on some respondents who self-identified with these groups;

*“Coming from an area of poverty myself it was disbarring to hear how individuals from poverty-stricken areas were spoken as though it is inevitable that they will have poor health, education, and low prospects.”*

Learners also discussed how their peers’ attitude to and treatment of curricular content and learning events impacted upon learners themselves. There were various reports of the interaction between these two features of respondents’ environments which created a mesosystem and had associated personal impacts on the respondent. Where peers were unaware of impacts of specific learning differences and near peer learning promoted non-inclusive practices, this appeared to impact on learners’ participation and sense of personal integration and belonging in that context.

*“Explain to your staff and students that peoples' pronouns are not something to joke about, I have had …coursemates joke or complain about them and it's very exhausting dealing with it.”*

*“There were some mental health CBL cases, but I feel there is still a lot of stigmatizing attitudes from other students.”*

Indirect linkages were also observed by unaffected students, who commented on the observed & reported effects on their peers.

*“I have also been told of my colleagues experiencing racism from facilitators and other students, one incident appeared to be a miscommunication but was still awkward for the student involved.”*

#### Multi-setting participation and Inter-setting knowledge; Differential expectations and experiences in different settings

Medical students by nature participate in multiple settings within the university and on clinical placements. The interactions between settings can cause disorientation.

*“I have witnessed a lot of misogynistic behaviour [at hospital site] this block which I was not sure how to deal with (until seeing the H5P [training]). This evolved around women not 'being suitable' for specific roles. I have looked to seek support for this from my tutor.”*

Information or experiences that exists in one setting about the other setting was evident in some responses which noted interactions, continuity or discrepancies between the practices advocated and noted in the medical school environment compared to those experienced in the clinical environment. In some instances, this resulted in dissonance.

*“A session on the topic of whether patients were allowed to refuse healthcare staff on the basis of ethnicity. At times it felt that racism was being justified on the basis of patient autonomy and I didn't necessarily have confidence…that should that situation occur in an NHS hospital, that I would be fully supported as a student....the NHS has a zero-tolerance policy against other forms of violence and abuse but it seemed that racism was not always considered a form of violence.”*

#### Inter-setting communication; differential expectations and experiences in different settings

Respondents described issues that had arisen in clinical learning environments. Instead of addressing these issues directly, they chose to raise these with the medical school via evaluation feedback or the designated raising concerns process. Students appeared to believe that the medical school had responsibility for its clinical teachers and their conduct and were willing to report back in some situations.

*“Experienced a fair amount of sexism in the trust. I think it might be worth training consultants affiliated with the uni …Staff should be told that excluding female students from learning opportunities…is sexist”.*

### Exosystems

Exosystems refer to factors that, although not within the learner’s immediate environment, still have influence on the learner’s experiences [30, 31]. Factors discussed here included university policies and procedures, the medical school curriculum and its delivery, financial struggles  and collective values held within staff and student communities and clinical settings.

#### Organisational commitment to EDI

Varying perspectives were noted in relation to the school leadership and faculty commitment and action in relation to creating an equitable, diverse, and inclusive curriculum. Numerous positive comments discussed improvements noted to aspects of the curriculum e.g., CBL cases, provision of active bystander training. These developments and other features of the school’s culture contributed to creation of a positive and inclusive learning culture and one that was committed to effecting positive change.

Despite positive interventions in this area, other respondents discussed where improvements were much needed. Continued neglect of these areas suggested failure by the school to uphold and deliver on their expressed commitment to EDI. Respondents cited instances where enhancements were largely tokenistic in nature.

*“I felt like a lot of the CBL cases attempted to be more inclusive by adding names and pronouns but that's where it stopped. It didn't feel like the case really addressed anything beyond that or health inequalities that could have been experienced by patients.”*

The mode of delivery of the medical curriculum was noted to be directly disadvantageous to students with specific learning differences.

*“The emphasis is all on reading and writing and very little on practical and visual. This is a global problem in education but would like to keep pointing out that this is fundamentally wrong ...and all learning styles should be catered for equally.”*

#### Overarching attitudes and awareness regarding EDI; faculty, peers, and clinicians

Respondents shared perspectives that suggested some faculty members were not demonstrating appropriate attitudes and suggested additional faculty training and development were required.

*“I feel that many staff members slip into the mistake of confusing man/woman and male/female. I think that, when discussing biological sex, the terms male/female/intersex are most appropriate, whereas man/woman represents a person's gender identity.”*

*“Dermatology lecture - Filled with white skin. Aside from 2 images of Afrocarribean skin. This should not be happening...Was quite shocking after the BLM movement…causing much discussion about decolonizing the curriculum that this lecture occurred.”*

Attitudes of other students within the learning community also impacted on the culture experienced by respondents, both positively and negatively.

*“Some students keep saying they don't understand the point of diversity, equality, and inclusivity training. This really surprises me as I thought fellow students would be more understanding.”*

*“There are quite a few people on our course that come from privilege[d] backgrounds that also say quite questionable stuff…some people were asking for evidence on the rates of death amongst black women even though the people giving the talk had referenced…. From this you could tell they don't think there is an actual race issue in the healthcare system.”*

Clinical settings exhibited implicit attitudes to mental health and wellbeing; impacts associated with these were also discussed by respondents;

*“I overheard a conversation around mental health and suicide that distressed me a fair bit, it was in the staffroom, but I found it inappropriate and had to remove myself. Making jokes about suicidal patients on myself and my CPs [*clinical partner*] first day in the practice, I’ve lost someone to suicide, and I felt incredibly upset****.”***

A feature regularly noted was the excusing of behaviour by labelling it as a joke and the suggestion that those being othered cannot take a joke.

#### Wider university policy and support for EDI

University policies for financial support and bursaries and their impacts on respondents were also discussed. Some financial support typically offered to undergraduates did not extend to this graduate-entry group and this policy served to reinforce and perpetuate economic barriers to effective participation in medical education. Similarly, programme structure and organisation also exacerbated these effects.

*“…the absolute lack of consideration for anyone needing to organise part time work (low income/low financial support groups) or childcare by being totally unorganised.”*

### Macrosystem

The macrosystem describes the social and political climate within which the educational environment sits [31, 32]. Many respondents referred to the wider societal drivers in relation to EDI and interpreted their experiences of the curriculum in this broader context. Respondents appeared to indicate awareness and personal commitment to this action. Occasional comments represented an exception to this observation, noting that efforts in this area were unnecessary, performative, and following a populist trend.

#### Societal and health inequalities, emerging evidence base

Respondents referred to emergent research agendas and findings regarding impacts of health inequalities, and the need for learning content to be broadly representative of patient populations. Respondents discussed issues of gender bias and historical exclusion of women from biomedical research, increased perinatal mortality amongst racially minoritized individuals and clinical manifestations of disease amongst racially minoritized individuals. Respondents expressed concern, frustration, and dissatisfaction where evolving knowledge and most current evidence base was not referenced in curricular content and teaching materials.

*“The fact that Black women are 5x more likely to die during childbirth was only mentioned in CBL - this should have had a much more central presence given the importance of the issue. I feel like on this occasion (programme) failed to deliver appropriate teaching. There was no mention of trans health issues or mentions of families outside the so-called nuclear model (e.g., single parents, LGBT*+ *families etc.).”*

*“The lecture on Gender, Ethnicity and Health felt outdated and tone-deaf…There was also no mention of important topics such as the research funding gap between perceived male and female health issues.”*

Respondents also highlighted circumstances in which established norms or traditional attitudes in medical education may contribute to and perpetuate societal and healthcare inequalities.

*“The session about STIs included some stigmatizing stereotypes about gay men including the use of sensationalist media headlines. I think when teaching about the gay community and sexual health it is important to acknowledge that harmful stigma that can create health inequalities.”*

#### Predominance of western culture

Efforts towards EDI were in some instances curtailed by established norms that adversely impacted inclusion of students or other stakeholders. Exclusively Western values and cultures appeared to determine aspects of learning experiences, and curriculum organisation and delivery. Examples included respondents’ reported difficulties in accessing annual leave for cultural or religious festivals. Respondents occasionally perceived hostility which they attributed to arising from their appearance or dress, resulting in differential and exclusionary treatment and a negative experience, as described by one respondent;

*“Braids were tied back off the collar on a ward, which I felt was within the dress code, I was told by the ward manager that I would have to tie them up. I didn't question this at the time, but was wondering how my braids being tied back differed to the pony-tail style adopted by many other staff.”*

*“I think that in clinical skills privacy screens should be provided. As a hijab wearing Muslim women, I feel that I have been made to feel a bit uncomfortable completing some of the examinations such as abdominal exams without the option to be screened from others.”*

#### Gender stereotypes and heteronormativity

Overarching gender stereotypes and gender-based exclusion were also evident. Career aspirations of female students were assumed by others to be impacted or limited by their gender and intentions towards having a family.

Males and racially minoritized males occasionally reported that these characteristics meant that they experienced barriers to learning opportunities in some specialties.

*“Being a brown male on the labour ward means be ready to be rejected or women to say they don’t want you there…. some women are uncomfortable. Males may be disadvantaged in this regard.”*

Respondents directly experienced non-inclusive behaviours. From respondents’ perspectives clinician educators also appeared to be less inclined to interact with or had lower expectations of students with particular (minority) characteristics e.g., female, racially minoritized students. This observation was reported directly by the those affected and elsewhere noted by peers.

Respondents reported that binary constructs of gender persisted in both curricular content and interactions with educators and clinicians. Similarly, heteronormativity was discussed as prevailing in some curricular content.

*“Everything was referred to as 'mum and dad' which I found slightly concerning considering as future doctors we have to be aware that the family unit will not always be a cisgendered man and woman. LGBTQ*+ *people going through pregnancy and parenting will face different hurdles and discrimination than cisgendered straight people & I feel this should have been acknowledged.”*

Conversely, however, other respondents appreciated the improvements and representativeness noted in some learning materials.

*“I enjoy the diversity in CBL cases and the inclusion of a gay couple without it being to do with HIV or overly fitting stereotypes.”*

### Chronosystem

The chronosystem reflects changes and patterns of environmental events and conditions over time.

#### Improvements (or lack thereof) over time

Some students discussed and expressed appreciation for improvements that they noted between blocks or over academic years. Other expressed frustration at lack of action and progress, with issues previously raised remaining unaddressed. These experiences appeared to undermine credibility of the school’s commitment to EDI and respondents’ faith in this work.

*“During this block many students have had to repeatedly bring up concerns about ensuring we see content in our sessions relating to presentation of different complaints on different skin tones. ...some students feel frustrated for having to continuously bring up this concern. It can appear that sometimes these concerns are not taken seriously.”*

### Preparation for future practice

Where respondents identified issues in relation to inadequate or inappropriate curricular treatment or coverage of EDI-related subjects, they regularly framed these observations with reference to their own preparedness for their future professional practice, forecasting ahead to their future professional practice. Respondents considered implications of non-inclusive or non-representative content or failure to acknowledge inequalities for patients and populations that they would serve and the impacts of early training experiences on their future professional identity and attitudes. Current medical students view these areas as being crucial to address from inauguration to medical training and not as optional supplements to other core content and offered several suggestions on how to do so.

*“I noted that there wasn't much of a focus on how BAME individuals are more susceptible to…CVD which I feel is important in clinical practice. Similarly, some blood conditions like sickle cell are much more common in people of colour, which I felt was not emphasised as much as it could have been.”* *“Pronouns were not really talked about in SocPop [Social and Population Perspectives]…I think this should be compulsory as doctors to learn to use correct pronouns to not alienate patients.”*

*“Mental health issues were treated very poorly…we're going to see patients, even colleagues, with mental health conditions from our first day…and it impacts on literally every other facet of health we've covered. Need to introduce students to it earlier and foster a healthier view - it's exactly why so many clinicians are scared of psych…”*

## Discussion

This study analysed a pooled dataset of student comments to explore student experiences of EDI in the curriculum and learning environment, to understand the factors contributing to these experiences and to attempt to identify underrecognised factors and influences here. Despite considerable interest and action in improving EDI within medical education, including at our medical school, significant issues still persist, and furthermore impactful interactions and curricular shortcomings have not always been widely recognised. Areas where enhancements have been implemented have undergone partial improvement, with scope for further travel. Implementation of this question has solicited comments describing multiple instances of poor practice, alienation, and exclusion, influenced by all levels of the ecosystem. Findings and analysis aid understanding of ongoing differential awarding of minoritized groups by revealing the ongoing prevalence of non-inclusive, inequitable practices. Routine opportunities to recollect and respond regarding EDI related issues garnered responses from individuals directly affected as well as precipitating empathetic reflections on possible impacts on others.

### Scale and scope of the problem

The question references protected characteristics stated in equalities legislation, signposting types of issues students may wish to consider, while also encouraging responses outside this framework. Many comments were situated directly in students’ own experiences and impacts arising from these experiences, suggesting that responses were authentic and not simply precoded according to the question, and that this question was effective in uncovering novel issues and underrecognised experiences (see Table 2). Recommendations for fostering inclusion in learning environments call for consistent opportunities to provide anonymous, unfiltered feedback [43] and transparent reporting of EDI data [2, 17] as a means to drive action and organisational accountability [17], though concerns about the ability to act on anonymous reports are described [44]. In this study, deficits in curriculum content and delivery (e.g., limited representativeness) and microaggressions in interpersonal interactions persist and, in some instances, are contrary to faculty assurance that curricular and environmental deficits have been resolved. Elsewhere, examples of outright exclusion were described e.g., clinicians’ preferential interaction with majority ethnic or male students, and educators’ selective use of only familiar student names (indirect linkage, mesosystem), illuminating the occurrence and, crucially, the influence and impacts of this previously-concealed experiences. This anonymous feedback question may give voice to those affected and unable to challenge subtle, accepted practices or more complex ethical or professionalism issues, thereby driving accountability and informing development of further interventions.

### Impact on learning & professional identify formation

Groups of students are being denied equitable educational experiences in both university and clinical settings. Measures towards inclusion often effectively require suppression of minority identities and cultures, and assimilation of dominant, privileged culture and practices [17], disrupting both professional identity formation and belonging [45–47], dynamics also highlighted in findings here. Negative experiences, including microaggressions, framing inappropriate behaviours as jokes, and direct and indirect exclusions are disruptive to students’ professional identity formation and belonging, as minoritized identities are devalued [8, 9, 43, 48]. Policies and procedures may fail to encompass informal, day-to-day occurrences that have significant impacts [49].

### Complexity and culture change

We applied Bronfenbrenner’s ecological systems theory as a framework to interpret findings in relation to EDI and associated influences on learners in their environment. This model vividly demonstrates the interplay and interdependency of different factors and systems, including those most entrenched and at risk of being overlooked. Modifications and intentions in the microsystem may fail in the absence of a congruent and supportive exosystem or macrosystem. Interactions and links between features of the microsystem can create mesosystems which may be hospitable or hostile. Seemingly remote features of the exosystem or macrosystem appeared to have significant impact on respondents. Driving change in such complex systems is not straightforward and requires cultural change. Schein [50] describes 3 levels of organisational culture- artifacts, espoused values, and basic assumptions. Much focus has been placed on addressing the artifacts (observed behaviours) and espoused values (set by the organisation); however, without addressing the underlying beliefs, significant change is unlikely to occur. Similarly, there is a tendency to address microsystem issues but, without addressing broader systemic issues and policies, change is restricted [51]. The cultural climate amongst the student population demonstrates some notable progressive features with peers identifying issues and expressing concern for others; there are opportunities to harness these student perspectives to develop faculty and the wider organisation.

Many respondents appeared to view awareness of and skills for EDI-related issues in healthcare as being among baseline competencies for graduating doctors, and consider that they will be accountable for meeting these requirements. Shortcomings in learning materials were considered in the context of future clinical practice and professional identities, and linked this to the existence of health inequalities within minoritized populations. Respondents readily recognised instances where teaching failed to reference emergent research findings and current evidence, demonstrating keen macrosystem awareness and perspectives, at times appearing to exceed that of educators. Medical education regulators have defined curricular outcomes expected of graduates, and these increasingly consider competencies in relation to EDI [5, 20, 21]. However, concerns have been noted regarding lack of specificity in authoritative sector guidance [52]. Respondents in this study had rich insights to offer regarding curricular and learning environment inclusivity by design. This finding, in the context of potentially ambiguous guidance for developing curricula to meet culturally diverse healthcare needs, further reiterates the need to capture and act on student voice.

### Everybody’s business

While all students have the opportunity to complete this “end of block” evaluation question, it is possible that those most personally affected by EDI-related issues would be most motivated to respond. However, it was clear in reviewing responses that comments were received from those personally affected by issues, as well as respondents contemplating impacts of exclusions on others including their peers (indirect linkage) or future patients (chronosystem). Uncertainty and speculation were also noted in some of the responses, suggesting developing reflexivity and awareness amongst students. Further, providing this opportunity regularly and consistently may train and habituate *all* students to consistently reflect on their experiences and identifying problematic practices or issues in their professional learning and practice environments, thereby also enhancing awareness and may promote perspective taking [45] and allyship [53] amongst groups who themselves may be personally unaffected by issues relating to EDI. Positioning these efforts as “everybody’s business” reduces the burden placed exclusively on minoritized groups to self-advocate and alleviates effects of the minority tax [18, 54].

### Strengths & limitations

The dataset included 376 comments, representing perspectives and experiences from students across the four programme years, and from university-based education and clinical placements. We were able to identify which phase or programme year students were commenting in relation to and noted that students in clinical years made fewer comments that students in the preclinical first year of the programme. Fewer responses from clinical students may be due to feelings of hopelessness (described in some of the comments), use of alternative routes to raise concerns or increasing confidence to use their allyship and active bystander training to intervene contemporaneously. Responses were received from both students personally affected by experiences and issues, and students contemplating impacts on others e.g., their peers or future patients. Due to the timeframe, it is not possible to track response patterns to this question across specific cohorts. This study timeframe may in turn have limited the ability to demonstrate issues occurring in the chronosystem. However, in relation to changes occurring over time, student comments noted improvements that they had observed and also regularly discussed and predicted their burgeoning and future professional responsibilities and role, as clinicians and considered the necessity of inclusive and evidence-based awareness, and how well their education experience contributed to this. In considering some mesosystems factors, these were challenging to distinguish between due to strong overlaps e.g., regarding multi-setting participation and inter-setting knowledge. Overall, however, application of EST allowed impacts of numerous factors at a range of levels to be clearly demonstrated, and illuminated the complexity of learning environments and factors impacting EDI.

This study considers pooled data collected regularly from only a single open-ended survey question. Limitations regarding richness of data and generalizability need to be considered in interpreting these findings and associated recommendations. The anonymous nature of this data collection method, exploring issues embedded in power structures and hierarchies, was felt to enable responses in an ethically acceptable way, not facilitated by other methods e.g., interviews, focus groups, hence the latter are not available for comparison. Data were triangulated against other sources, namely issues reported by students in the raising concerns process. Students are influenced by their ongoing experiences and interactions within educational organisations in deciding on whether to report a concern [42, 55]. Students may not always feel enabled or safe to report concerns regarding problematic issues they have encountered, and this is particularly true in the case of minoritized students, where perceived power differentials between learner and the organisation are even greater. The anonymous evaluation questionnaire may overcome some barriers to reporting negative experiences, as it reaches *all* learners regularly as part of routine student feedback on learning experiences. Similarities and differences between the two sources were noted; overlap between the nature of discrete issues raised (e.g., in relation to microaggressions) were clear and expected. As intended, more severe or urgent issues were sometimes reported directly via the designated and rapidly responsive reporting concerns process, rather than respondents waiting until the end-of-block feedback form was available to report a concern. In other cases, respondents indicated that they had simultaneously reported via the programme’s raising concerns process, indicating that some more severe concerns were also captured within this data set. Novel and previously unknown issues not captured by raising concerns were surfaced, as were issues not reaching the threshold to be considered by students as a concern – a well-described dilemma experienced by students [56] Responses to the feedback question were at times contemplative and speculating, with students indicating that the question had stimulated them to reflect on their experiences and consider potential impacts and experiences of their peers.. Respondents articulated uncertainty as well as praise for efforts in curricular enhancements and positive interpersonal interactions, while the reporting concerns process which does not capture experiences of satisfaction or exemplary positive practices or interactions. In considering some mesosystems factors, we identified a close overlap between e.g., multi-setting participation and inter-setting knowledge. However, EST allowed impacts of interactions and environmental factors to be more clearly demonstrated than by raising concerns reports. These measures and resulting observations support the ecological validity of this process. Future exploration of updated datasets from more recent student cohorts may identify evidence of improvements in student experience of EDI or reductions in number or severity of issues reported. Alternatively, as awareness of EDI-related issues continues to expand, increasing numbers and types of issues may continue to be identified.

Our own positionality as two white female researchers was considered and a range of steps taken to address limitations here, including perspective taking from faculty leadership experienced in inclusive education and from students, described earlier. These data were triangulated against other sources, namely issues reported by students in the raising concerns process.

We explored the commonly recognised construct of EDI as an aspiration towards fairer societies and organisations, employing *Equality,* an approach that contrasts with other geographical settings where “Diversity, Equity and Inclusion” are pursued. We recognize this equity is rightly preferred in some policies and strategies as it avoids biases that occur with equality [57], and adoption and understanding of this entity and associated terminology in our setting is recommended as a future ambition.

Our analysis noted that experiences in “university-situated” education may differ from those in clinical environments and could have been analysed separately. Issues noted during didactic teaching often related to content and presentation of learning materials, whereas in the clinical environment interpersonal interactions with staff and patients predominated. However, we did not wish to imply differential standards for these respective settings. Furthermore, EST has been used to effectively demonstrate the complexity of interaction between individual, social and cultural factors in interprofessional learning [29] and has been applied to inform insights into complex, multi-setting, professional learning environments [31]. We advocate sharing data, experiences, and EDI goals with clinical education partners to ensure a cohesive approach. Further, we hope that future graduates will continue to habitually pursue equality, diversity, and inclusion in their professional environment. Patient and educator perspectives merit exploration and implementation in ensuring holistic consideration of inclusion, representativeness, and fairness in medical education.

### Implications for healthcare education

To fulfil sector commitments to equality, diversity, and inclusivity, further understanding of systems contributing to learner experiences and environments, and associated impacts on learners must be established. Structural exclusions and microaggressions are, by definition, subtle and are  longstanding accepted and tolerated norms. Enhanced, sustained efforts are required to identify such issues, which may have wide-ranging impacts across the learning environment. Understanding direct and indirect impacts at different ecological levels can shift away from deficiencies-based approaches and instead help determine a systematic approach to structural and cultural change [5, 30, 51]. Measures to empower student voice, as described here, in addition to responsiveness to addressing feedback are recommended to ensure EDI agendas can be fulfilled and belonging in education and training guaranteed for all.

### Supplementary Information

**Additional file 1.**

## Data Availability

The datasets used and/or analysed during the current study are available from the corresponding author on reasonable request**.**

## Abbreviations

BAME: Black, Asian and Minority Ethnic
BLM: Black Lives Matter
BME: Black and Minority Ethnic
CBL: Case based learning
CVD: Cardiovascular disease
DEI: Diversity, equity and inclusion
EDI: Equality, diversity and inclusion
EST: Ecological systems theory
MBChB: Primary medical degree qualification awarded in the UK
NHS: National Health Service, UK
