# Supplementary material for: Medical student experiences of equality, diversity, and inclusion: content analysis of student feedback using Bronfenbrenner’s ecological systems theory
Source: BMC Med Educ. 2024 Jan 3;24:5. doi: 10.1186/s12909-023-04986-8 (PMC10765790; doi:10.1186/s12909-023-04986-8)
Supplement: Supplementary file 1 — Additional file 1. [file 12909_2023_4986_MOESM1_ESM.docx]

| Code Name | Code Description |
| --- | --- |
| **Accommodations/ Reasonable Adjustments** | Comment discussed accommodations or adjustments for learners with a disability or specific learning difference, usually for assessments. |
| **Belonging** | Sense of integration and acceptance in the learning environment and organisation |
| **Changes over time** | Comment discussed change or lack of change occurring over time in relation to EDI issue. This may include that feedback not addressed or that there was a lack of response or action in relation to learners’ feedback. |
| **Clinical environment** | Experience or issue commented on relating to EDI pertained to or occurred in a clinical learning environment |
| Differential expectations and knowledge of clinical environment vs. in medical school/ university | Comment indicated that respondent held differential expectations of e.g., attitudes, behaviours, ethos in clinical environment vs. in medical school/ university or had differential experiences in the clinical environment vs. in medical school/ university, particularly relating to EDI. |
| Experiences with clinicians | Comment discussed experiences with clinician(s) which related to an EDI issue |
| Experiences with patients | Comment discussed experiences with patient(s) which related to an EDI issue |
| **Curricular content** |  |
| Current content | Content is up to date, in line with most current evidence and recommended practice |
| Curricular content not representative | Content is not representative of diverse patients and populations. Includes visual representation e.g., in dermatology teaching, and depictions of patient social contexts and backgrounds e.g., in case-based learning. |
| More content on EDI | Additional content relating to and focus on issues relating to EDI, usually recommending this. |
| **Curriculum; aspect of curriculum or subject area** | Aspect of the curriculum/ course content discussed in comment and how this related to EDI. |
| Anatomy |  |
| Case based learning |  |
| Clinical skills |  |
| Community Learning |  |
| Dermatology |  |
| Social and Population Perspectives theme |  |
| **Disability or Specific Learning Difference** | Experience of disability – personal or that of peers, that may have implications for learning. |
| **Diversity** | Experience or issue commented on or pertained to diversity |
| **Ethnicity** | Experience or issue commented on or pertained to ethnicity |
| **Experiences with educators** | Comment discussed educator and their perceived attitude to or treatment of EDI related issue |
| **Faculty and faculty development needs** | Faculty knowledge, skills, and attitudes, usually perceived as relating to issues or aspects of EDI. |
| **Faculty and medical school leadership** | Faculty and medical school leadership and how leadership approaches, strategies and attitudes impacted on EDI |
| **Faith, religion** | Experience or issue commented on or pertained to faith or religious beliefs. |
| **Gender/ LGBTQIA+ issues** | Experience or issue commented on or pertained to gender minorities or issues experienced by or relating to LGBTQIA+ individuals or groups |
| **Health inequalities** | Comment discussed health inequalities impacting patients or populations, including how these were addressed or exploring learning content or context |
| **Inclusion** | Experience or issue commented on or pertained to inclusion. |
| **Inclusion by design** | Experience or issue commented on or pertained to inclusion by design, usually in relation to learning resources. Inclusion of diverse learners has been considered in initial development or design, reducing the need for later, retrospective adaptations or adjustments. |
| **Indirect linkage** | Experience or issue commented on elements interacting with each other, that have indirect impact on learner. |
| **Language, terminology** | Comment discussed use of language or use of dated or non-inclusive language or terminology. |
| **Learning resource(s)** | Experience or issue commented on or pertained to learning resource(s). |
| **Medical school and university policy and practice** | Comment discussed medical school and university policy and practice and how this impacted on an aspect of EDI |
| **Mental health** | Experience or issue commented on or pertained to mental health |
| **Microaggression** | Experience or issue commented on or pertained to reported or described indirect, subtle, or unintentional discrimination against member(s) of a marginalised/ minoritised group. |
| **Names** | Experience or issue commented on or pertained to use of individual’s correct name and correct pronunciation |
| **Weight stigma/ body image** | Experience or issue commented on or pertained to weight stigma, body image, fat shaming |
| **Peers; attitudes, interactions support** | Experience of or issue relating to support from peers (fellow students) |
| **Personal, emotional impact** | Emotional impact or feelings described or expressed in relation to experience, or issue reported. May include respondents’ own emotional impacts or those observed in peers. |
| **Policy and practice in medical education sector** | Comment discussed medical education and training sector policy and practice and how this impacted on an aspect of EDI |
| **Political correctness, performativity** | Responses suggesting that efforts and action in relation to EDI were rooted in excessive “political correctness” and appeared performative or populist. |
| **Positive feedback** | Comment expressed appreciation or praise for a positive experience (or an improvement noted usually relating to an aspect of EDI) |
| **Prepared for practice** | Comment discussed whether or not learning experience, curricular content ensured preparedness for future professional practice, usually with reference to EDI as impacting on patients and populations and healthcare context. |
| **Raising Concerns** | Comment referred to raising/ reporting concerns. May include that the comment had already been shared via the Raising Concerns process *or* that comment wasn’t being provided in response to the question, as the issue had already been highlighted via the Raising Concerns process |
| **Sexism/ gender stereotypes** | Issue/ experience mentioned included element of observed sexism or experience of gender stereotyping. |
| **Socioeconomic inequalities or hardship** | Experience or issue relating to socioeconomic background, inequalities and associated impacts, financial hardship, or financial support available. Comment may concern patient, population, or the student. |
| **Western cultures** | Comment discussed predominance of western culture and how this impacted on an aspect of EDI |
| **Suggestions for changes or improvements** | Respondent’s suggestion for how an issue could be addressed or further improvements could be made to enhance aspect of EDI. |
| **Unsure** | Indicating that they are reflecting on possible issues, considering peers’ perspectives and experiences during the block (or placement). |

Codes are indicated in **bold.** Subthemes are indicated underneath the code and in normal font.
